# Aberrant dynamic minimal spanning tree parameters within default mode network in patients with autism spectrum disorder

**DOI:** 10.3389/fpsyt.2022.860348

**Published:** 2022-09-15

**Authors:** Huibin Jia, Xiangci Wu, Zhiyu Wu, Enguo Wang

**Affiliations:** ^1^Institute of Psychology and Behavior, Henan University, Kaifeng, China; ^2^School of Psychology, Henan University, Kaifeng, China; ^3^Huaxian People's Hospital of Henan Province, Anyang, China

**Keywords:** autism spectrum disorder, default mode network, dynamic functional connectivity, minimal spanning tree, graph theory

## Abstract

The altered functional connectivity (FC) level and its temporal characteristics within certain cortical networks, such as the default mode network (DMN), could provide a possible explanatory framework for Autism spectrum disorder (ASD). In the current study, we hypothesized that the topographical organization along with its temporal dynamics of the autistic brain measured by temporal mean and variance of complex network measures, respectively, were significantly altered, which may further explain the autistic symptom severity in patients with ASD. To validate these hypotheses, the precise FCs between DMN regions at each time point were calculated using the resting-state functional magnetic resonance imaging (fMRI) datasets from the Autism Brain Imaging Data Exchange (ABIDE) project. Then, the minimal spanning tree (MST) technique was applied to construct a time-varying complex network of DMN. By analyzing the temporal mean and variance of MST parameters and their relationship with autistic symptom severity, we found that in persons with ASD, the information exchange efficiencies between cortical regions within DMN were significantly lower and more volatile compared with those in typical developing participants. Moreover, these alterations within DMN were closely associated with the autistic symptom severity of the ASD group.

## Introduction

Autism spectrum disorder (ASD) describes a group of neurodevelopmental disorders, which could impair social communication ability and lead to restricted and repetitive patterns of behaviors or interests (1). The prevalence rate of ASD has increased drastically in recent years world over and ASD has become one of the global public health problems affecting human mental health seriously (2, 3). Thus, it is imperative to elaborate on the pathological mechanisms and potential risk factors related to ASD and develop efficient therapies.

With the recent development of modern neuroimaging techniques, particularly functional magnetic resonance imaging (fMRI), the exploration of the neuropathological of ASD has become possible. The functional connectivity revealed by temporal dependency between functional signals of distinct cortical regions could provide a possible explanatory framework for ASD (4). A theory of cortical hypoconnectivity in the autistic brain predicts that the interregional collaboration and information exchange required both in cognitive tasks and task-free resting-state (RS) would be underserved in ASD (5, 6). This theory has been supported by numerous neuroimaging studies, although cortical hyperconnectivity has also been detected in some studies (7, 8). Among all these functional connectivity studies, the hypoconnectivity involving regions of the default mode network (DMN) which are crucial to the normal development of social cognition abilities (e.g., self-representation, mentalizing ability, and emotion recognition) has been consistently observed in previous studies (9, 10). By representing the brain as a complex network with a set of nodes (i.e., cortical regions/scalp channels) connected by edges (i.e., neural connectivity), one can study its topographical organization by using concepts from graph theory to evaluate various graph parameters (11). Related research shows that the node-level and network-level complex network parameters are significantly altered in patients with ASD and could predict their autistic symptom severity, which suggested that the topographical configuration of the cortical network within the autistic brain tends to deviate from the optimal network organization (12, 13).

However, previous studies focusing on neural connectivity and topographical organizations between cortical regions of persons with ASD had certain limitations. For example, the widely used static functional connectivity techniques are too simplistic to elaborate all the temporal-spatial information of cortical activities, since previous research proved that temporal coupling between cortical regions fluctuated over time, even during task-free resting-state (RS) (14). In Jia et al. (15), the dynamic conditional correlation (DCC), which could assess the dynamic functional connectivity (dFC) between cortical regions and is a technique with high test-retest reliability, was applied to investigate the DMN-FC (i.e., the functional connectivity between DMN regions) patterns of persons with ASD. They showed that compared to the typical developing (TD) group, the ASD group exhibited a significantly lower temporal mean of DMN-FC and significantly higher temporal variance of DMN-FC. In our study, we intend to examine the topographical properties of the time-varying complex brain network of persons with ASD through the DCC technique and minimal spanning tree (MST) using the resting-state fMRI datasets of the Autism Brain Imaging Data Exchange (ABIDE), which includes functional and structural brain imaging datasets of more than 1,000 participants (16). The MST is a unique acyclic subgraph that contains the strongest connections from the set of all available weighted connections (17). As a network construction technique, one of the excellent features of the MST approach is that MST is unaffected by the thresholding problem (18). Moreover, recent studies have shown that MST analysis is effective and sensitive to capturing alterations of the topographical organization due to group differences and conditional effects in both functional and structural imaging datasets (17, 19, 20). We hypothesized that compared to TD participants, persons with ASD should exhibit significantly altered topographical organization measured by temporal mean and variance of MST parameters. Moreover, these abnormalities should be significantly associated with the autistic symptom severity in the ASD group.

## Materials and methods

### The resting-state FMRI datasets

The ABIDE project includes the resting-state fMRI (RS-fMRI) datasets, MRI datasets, and phenotypic information (including gender, age at fMRI scan, sex, IQ, and diagnostic information) of 1,112 participants (539 persons with ASD and 573 TD participants recorded by 17 institutions (so-called “*sites*”) (16). The data collection were conducted following the basic principles of the Helsinki declaration and were approved by the research ethics committees of these institutions. Informed consent was obtained from the participants or their legal guardians. Details of acquisition and experimental protocols of all the sites are available at http://fcon_1000.projects.nitrc.org/indi/abide/abide_I.html.

The RS-fMRI datasets of 343 persons with ASD and 428 TD participants were selected for the complex network analysis. The dataset selection was performed according to the following criteria: (1) datasets with MRI images providing near-full brain coverage and successful registration; (2) datasets passing manual quality assessments of three independent raters; (3) datasets with mean framewise displacement (*func_mean_fd*) < 0.2 mm; (4) datasets with percent framewise displacement >0.2 mm (*func_perc_fd*) < 25%; (5) individuals with IQ score >75; (6) individuals in ASD group with reliable diagnostic information obtained *via* Autism Diagnostic Observation Scale (ADOS) or Autism Diagnostic Interview-Revised (ADI-R); (7) datasets from sites with more than three participants in each group after selecting datasets based on the above six criteria (15).

The independent *t*-tests with groups (ASD group vs. TD group) as the independent variable and phenotypic information (i.e., age at fMRI scan and IQ) and image parameters (i.e., func_mean_fd and func_perc_fd) as dependent variables did not detect any significant group difference (*ps* > 0.05). For more information on the demographic data of these participants, see Supplementary Table S1.

### Resting-state FMRI datasets preprocessing

In this study, the fMRI datasets were preprocessed by the Data Processing Assistant for Resting-State fMRI (DPARSF) (21, 22), which is a convenient plug-in software based on Statistical Parametric Mapping (SPM) package and Resting-State fMRI Data Analysis Toolkit (REST) (23, 24). The image preprocessing consists of the following steps (15):

1. The first 4 image volumes within each fMRI dataset were discarded.
2. All volume slices were corrected for different signal acquisition times by shifting the signal measured in each slice relative to the acquisition of the slice at the mid-point of each TR.
3. The images for each fMRI dataset were realigned using a six-parameter (rigid body) linear transformation with a two-pass procedure (registered to the first image and then registered to the mean of the images after the first realignment).
4. Individual structural images (T1-weighted MPRAGE) were co-registered to the mean functional image after realignment using a six degrees-of-freedom linear transformation without re-sampling.
5. The transformed structural images were segmented into gray matter (GM), white matter (WM), and cerebrospinal fluid (CSF) (25). The Diffeomorphic Anatomical Registration Through Exponentiated Lie algebra (DARTEL) tool was used to compute transformations from individual native space to MNI space (26).
6. The Friston 24-parameter model was used to regress out head motion effects from the realigned data (27).
7. The signals from WM and CSF were regressed out to reduce respiratory and cardiac effects. Since previous studies showed that global signal regression (GSR) could yield substantial increases in negative correlations, GSR was not performed (28).
8. The images were registered in Montreal Neurological Institute (MNI) space with 3 mm^3^ cubic voxels by using transformation information acquired from DARTEL. The images were further smoothed by a kernel of 6 mm.
9. Temporal filtering (0.01–0.1 Hz) was performed on the time series to remove low-frequency drifts and high-frequency noise from the signal.
10. According to Andrews-Hanna et al. (29), 18 sphere regions of interest (ROIs) with a radius of 10 mm within DMN were defined. The centroid coordinate of each sphere ROI is shown in Table 1. The signal time series of each ROI was computed as the mean value of voxels within this ROI.

**Table 1:** The 18 DMN ROIs defined in the current study.

**ROI**	**Abbrev**.	**MNI coordinate**		
		***x***	***y***	***z***
**PCC-aMPFC Core**				
Anterior medial prefrontal cortex (ROI #1)	aMPFC	−6	52	−2
Posterior cingulate cortex (ROI #2)	PCC	−8	−56	26
**dMPFC Subsystem**				
Dorsal medial prefrontal cortex (ROI #3)	dMPFC	0	52	26
Left temporal parietal junction (ROI #4)	lTPJ	−54	−54	28
Left lateral temporal cortex (ROI #5)	lLTC	−60	−24	−18
Left temporal pole (ROI #6)	lTempP	−50	14	−40
Right temporal parietal junction (ROI #7)	rTPJ	54	−54	28
Right lateral temporal cortex (ROI #8)	rLTC	60	−24	−18
Right temporal pole (ROI #9)	rTempP	50	14	−40
**MTL Subsystem**				
Ventral medial prefrontal cortex (ROI #10)	vMPFC	0	26	−18
Left posterior inferior parietal lobule (ROI #11)	lpIPL	−44	−74	32
Left retrosplenial cortex (ROI #12)	lRsp	−14	−52	8
Left parahippocampal cortex (ROI #13)	lPHC	−28	−40	−12
Left hippocampal formation (ROI #14)	lHF^+^	−22	−20	−26
Right posterior inferior parietal lobule (ROI #15)	rpIPL	44	−74	32
Right retrosplenial cortex (ROI #16)	rRsp	14	−52	8
Right parahippocampal cortex (ROI #17)	rPHC	28	−40	−12
Right hippocampal formation (ROI #18)	rHF^+^	22	−20	−26

### Computing dynamic functional connectivities between ROIs

The dynamic functional connectivities (dFCs) between ROIs were assessed through the DCC method, which possesses at least the following advantages (14, 15). Firstly, unlike the traditional sliding-window (SW) based techniques, it does not need to choose a window length which is usually arbitrarily decided by researchers in SW-based techniques and could provide a functional connectivity level at a specific time point instead of a time window in SW-based techniques. Secondly, the test-retest reliability of the metrics provided by the DCC method is much higher than those of the SW techniques (30).

Before computing the DCC, each ROI signal with length T should be converted to a mean zero time series. Assume *y*_*t*_ is the converted time series of an ROI pair with dimension 2×T. The dynamic correlations *R*_*t*_ with dimension 1×T can be calculated using the following equations:


(1) $$\begin{array}{r} \sigma_{i,t}^{2}=\omega_{i}+\alpha_{i}\;\cdot \;y_{i,t-1}^{2}+\beta_{i}\;\cdot \;\sigma_{i,t-1}^{2},where\;i\;equals\;1\;or\;2 \end{array}$$

(2) $$\begin{array}{r} D_{t}=diag\left\{\sigma_{1,t}\;,\;\sigma_{2,t}\right\} \end{array}$$

(3) $$\begin{array}{r} \varepsilon_{t}=D_{t}^{-1}\;\cdot \;y_{t} \end{array}$$

(4) $$\begin{array}{r} Q_{t}=(1-\theta_{1}-\theta_{2})\;\cdot \;Q+\theta_{1}\;\cdot \;\varepsilon_{t-1}\;\cdot \;{\varepsilon^{\prime }}_{t-1}+\theta_{2}\;\cdot \;Q_{t-1} \end{array}$$

(5) $$\begin{array}{r} R_{t}=diag{\left\{Q_{t}\right\}}^{-1/2}\cdot Q_{t}\;\cdot \;diag{\left\{Q_{t}\right\}}^{-1/2} \end{array}$$

Firstly, each ROI time series within *y*_*t*_ is modeled by a generalized autoregressive conditional heteroscedastic (GARCH) model, which expresses the conditional variance of a single time series at time t as a linear combination of past values of the conditional variance and of the squared process itself [equation (1)]. Secondly, the standardized residual ε_*t*_ was computed through equation (2) and equation (3). Thirdly, the non-normalized version of the time-varying correlation matrix *Q*_*t*_ was computed using an exponentially weighted moving average (EWMA) window [equation (4)]. Note that, in equation (4), *θ*_1_ and *θ*_2_ are non-negative scalars satisfying 0 < *θ*_1_ + *θ*_2_ <1, $\bar{Q}$ can be calculated as $\bar{Q}=\frac{1}{T}\;\sum_{t=1}^{T}\varepsilon_{t}\;•\;\varepsilon_{t}^{T}.$ Lastly, *Q*_*t*_ is rescaled, which creates the dynamic correlations *R*_*t*_.

### Constructing time-varying complex networks using minimal spanning tree (MST)

After the dFCs of all ROI pairs were assessed through the approach illustrated above, we obtained a time-varying correlation matrix *C*_*t*_ with dimension *N* × *N* × *T*, where N and T were the number of ROIs and time points, respectively. *C*_*t*_(*i, j, k*) indicates the FC between ROI *#i* and ROI *#j* at time point k.

In this study, we used minimum spanning tree (MST) analysis to build the time-varying complex network within DMN. The MST consists of the strongest connections in the entire weighted undirected graph so that the sum of the weights of the edges (i.e., the reciprocal of DCC at a specific time point) included in the tree is minimized without forming cycles (17, 18). The MST is a connected graph without loops between nodes (i.e., the 18 ROIs within DMN) and without isolated nodes (i.e., there exists a path between each pair of nodes in the graph). An MST with N nodes has exactly *N*-1 edges/connections. Assuming the number of nodes is *N*, the MST at each time point was produced using Kruskal's algorithm in the following manner (31). Firstly, all the connections at a given time point were ranked from lowest to highest “weight”. As discussed above, the “weight” was calculated as the reciprocal of connection strength, and thus can be considered as the cost of information exchange between nodes. Secondly, after all the connections were removed, the connection with the lowest weight was added. Then, the connection with the second lowest weight was added and this procedure was repeated until all nodes were connected. If adding a new connection resulted in a cycle or loop, this connection was discarded, and the next connection ranked by weight was added to the graph. Lastly, the graph thus constructed was binarized (i.e., the existing edges and non-existing edges were given a value of 1 and 0 respectively).

### Computing the MST parameters

After the MST at each time point was constructed, the following parameters of each MST were computed (17–20).

1. The degree of certain node *k*_*i*_ (where *i* = 1, …, *N*). It is defined as the number of connections or edges for a given node. The nodes with high degree are “*hub nodes*” in the MST and are crucial to the information exchange between distinct nodes.
2. The degree of certain MST *K*. It is the degree of the node with maximum degree: *K* = *max*(*k*_*i*_).
3. The leaf fraction of certain MST *L*_*f*_. The “*leaf* ” refers to the node with only one connection. The leaf number in an MST ranges between 2 (a line-topology) and a maximum value *N* − 1 (a star-like topology). Leaf fraction is the actual leaf number divided by the maximum possible leaf number: *L*_*f*_ = *L*/(*N* − 1), where *L* is the actual leaf number in the MST. Thus, the leaf number, together with the leaf fraction, could be used to describe to what extent the MST has a central organization. A high value of leaf number or leaf fraction indicates that the communication within the network is largely dependent on hub nodes.
4. The assortativity coefficient of certain MST *R*. It equals the Pearson correlation coefficient of the degrees of two nodes at the end of connections, and ranges between−1 and 1. If 0 < *R* < 1, the MST is an assortative network, otherwise, it's a disassortative network.
5. The diameter of certain MST *D*. It is defined as the maximum value among lengths of all the available shortest paths. In MST, the shortest path between two nodes is defined as the path with least connections, and its length is the number of connections within this shortest path.
6. The betweenness centrality of certain node *BC*_*i*_ (where *i* = 1, …, *N*). It is defined as the total number of shortest paths between any two nodes that are passing node *i*, divided by the total number of shortest paths in the network: $BC_{i}=A/C_{N}^{2}$, where *A* is the total number of shortest paths that are passing node *i*. Nodes with a high *BC* are considered “*hub nodes*” not based on their number of connections, but on their importance for global communication in the network.
7. The betweenness centrality of certain MST *BC*. It is the maximum value of nodal betweenness centrality: *BC* = max(*BC*_*i*_). It could also be used to describe the global network organization of MST. In star-like topography, the *BC* value of MST equals to 1.
8. The global efficiency of certain MST *E*. It measures the average inverse shortest path length in the network, and thus relates to the efficiency of information exchange within the network.
9. The characteristic path length of certain MST *L*. It refers to the average of all the shortest path lengths in the network and could be used to assess the cost of information transfer within the network.
10. The degree divergence of certain MST κ. It measures the broadness of the degree distribution and could be calculated using the following formula: $\kappa =\frac{\langle k^{2}\rangle }{\langle k\rangle }$, where *k* is the degree of nodes. This measure is believed to be related to resilience against attacks on the network. Higher κ indicates a broader degree of distribution and higher vulnerability for targeted attacks.
11. The tree hierarchy of certain MST *T*_*h*_. It characterizes the balance between network integration and overload of central nodes and can be computed using the following formula: $T_{h}=\frac{L}{\left(2∙M∙BC\right)}$, where *L*, *M*, and *BC* are the leaf number, edge/connection number (i.e., *N*−1), and betweenness centrality of the MST.
12. The eccentricity of node *ecc*_*i*_ (where *i* = 1, …, *N*). It is defined as the longest distance between a certain node and the other nodes. Nodes with lower eccentricity are more prone to be hub nodes within the network.

### Computing summary statistics of MST parameters

**Figure 1:**
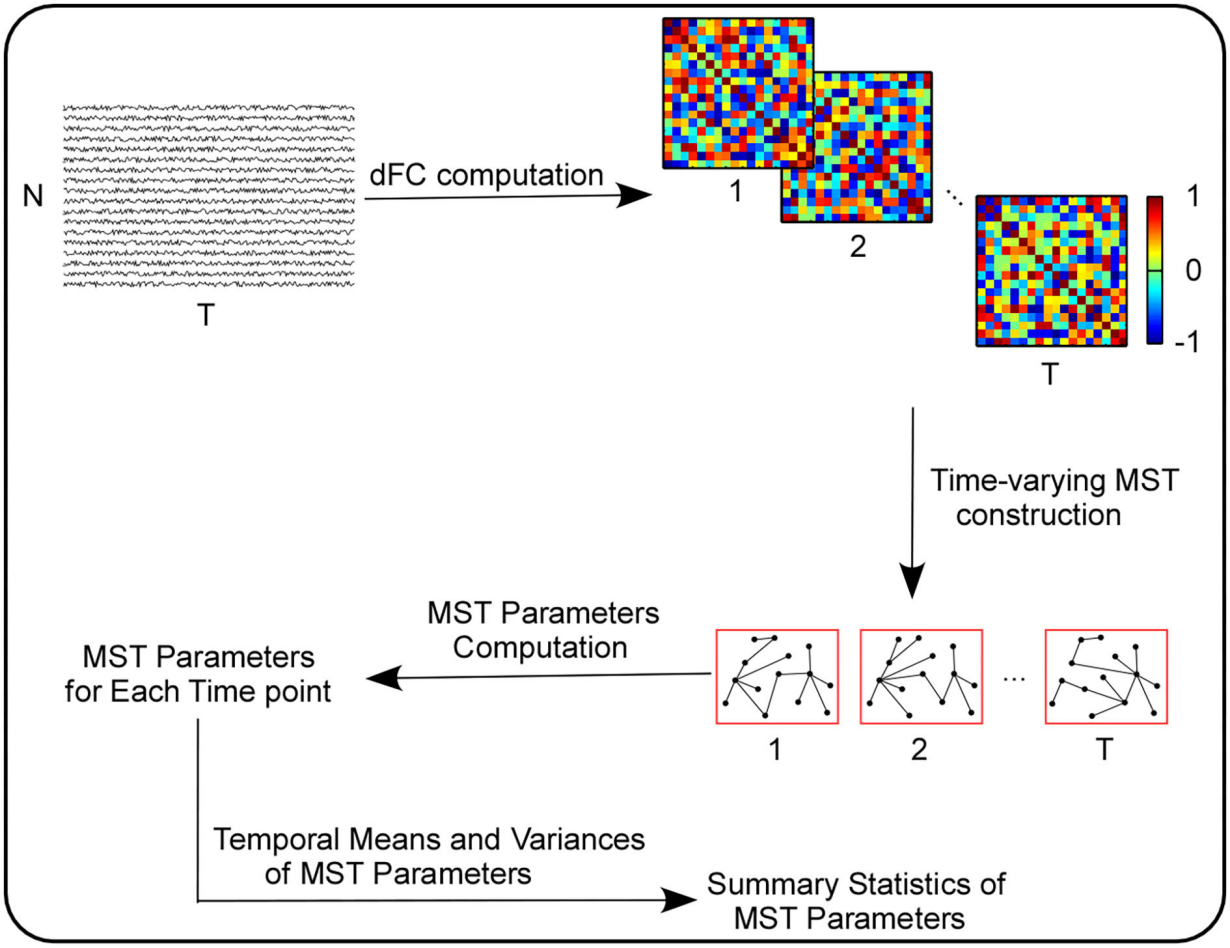
The computation pipeline of the summary statistics of MST parameters.

After all the network-level parameters (i.e., *K, L*_*f*_, *R, D, BC, E, L*, κ and *T*_*h*_) and node-level parameters (i.e., *k*_*i*_, *BC*_*i*_, *ecc*_*i*_, where *i* = 1, …, *N*) were evaluated for the MST of each time point, we computed two basic summary statistics for each parameter above, i.e., their temporal means and temporal variances. More information about summary statistics computation can be seen in Figure 1.

### Statistical tests on summary statistics of MST parameters

For the temporal mean and variance of network-level parameters and node-level parameters, independent *t-*tests were conducted with groups (ASD group vs. TD group) as independent variables, after controlling the effects of the following variables: the age at scan, func_mean_fd, func_perc_fd, IQ score, gender, and sites. To control the multiple comparison problem when testing the summary statistics of node-level MST parameters, the false discovery rate (FDR) procedure was used (32). The threshold for significance was *p* < 0.05.

### Correlations with autistic symptom severity

To investigate the relationship between the two summary statistics of MST parameters and scores of autistic symptom severity in the ASD group, the Pearson correlation coefficients between the two summary statistics of MST parameters and symptom severity as assessed by the ADOS total (ADOS_TOTAL), communication (ADOS_COMM), social (ADOS_SOCIAL) and stereotyped behavior (ADOS_STEREO_BEHAV) scores were calculated after controlling the effects of following variables: the age at scan, func_mean_fd, func_perc_fd, IQ score, gender, and sites. The significance of the correlation coefficients was assessed with a *t-*statistic. The threshold for significance was *p* < 0.05.

## Results

### Group differences in the temporal mean and variance of MST parameters

**Figure 2:**
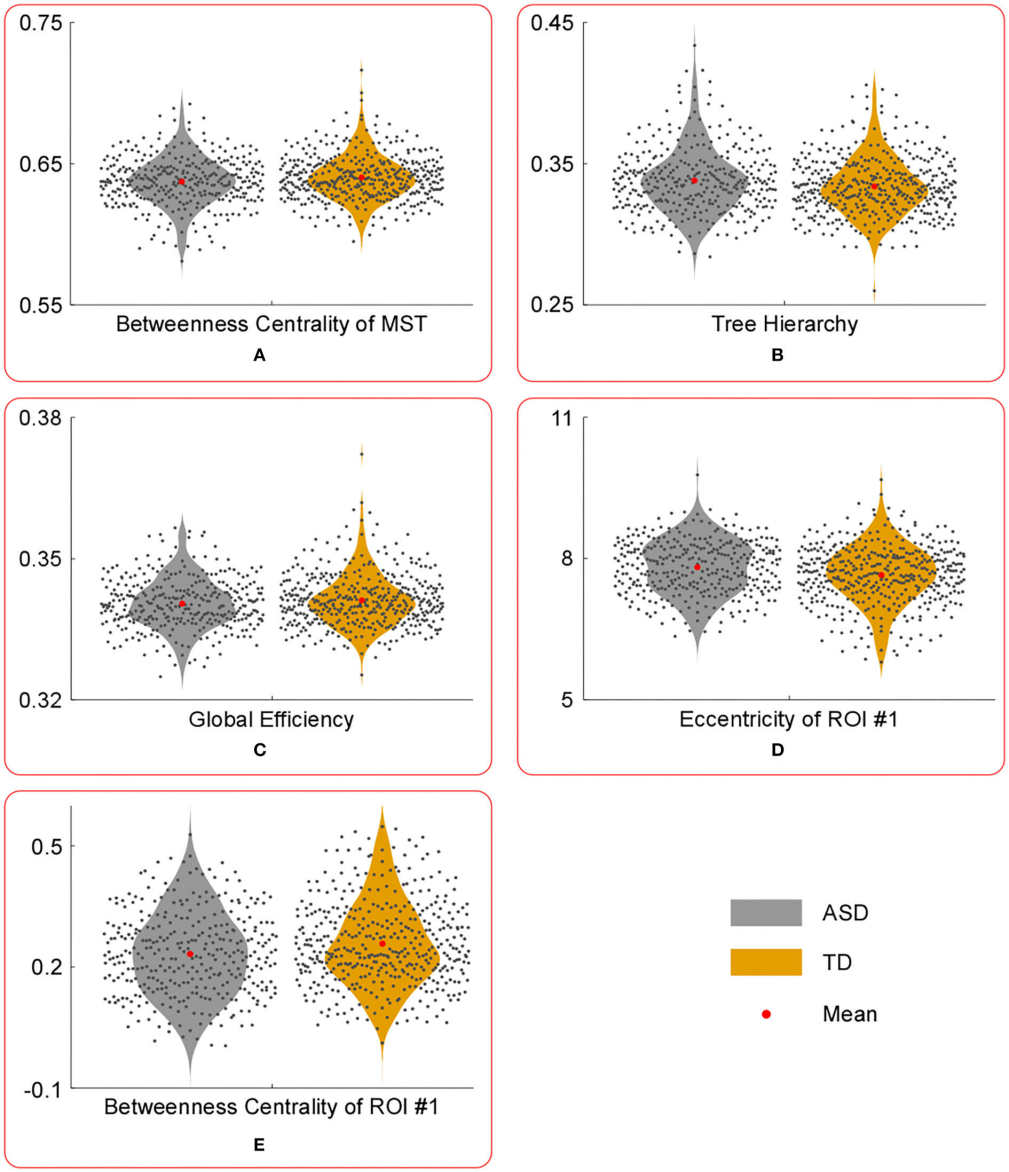
The violin plots of the temporal mean of MST parameters with significant group differences. Compared with those in TD group, the temporal mean of betweenness centrality of MST **(A)**, global efficiency **(C)** and betweenness centrality of ROI #1 **(E)** were significantly lower in ASD group, whereas the temporal mean of tree hierarchy **(B)** and eccentricity of ROI #1 **(D)** were significantly higher in ASD group.

**Figure 3:**
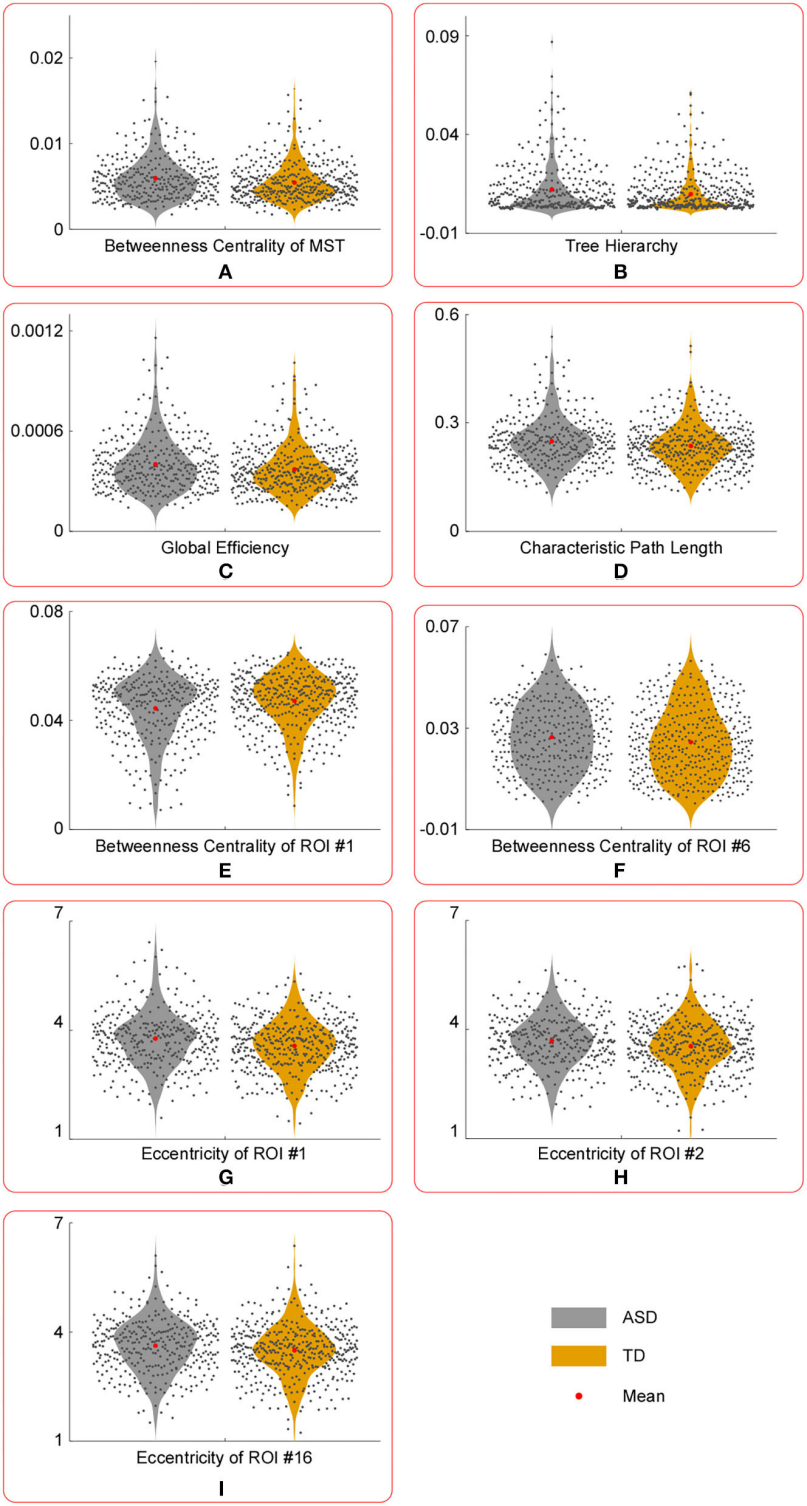
The violin plots of temporal variance of MST parameters with significant group differences. Compared with those in TD group, the temporal variance of betweenness centrality of ROI #1 **(E)** was significantly lower in ASD group, whereas the temporal variance of the following measures were significantly larger in ASD group: betweenness centrality of MST **(A)**, tree hierarchy **(B)**, global efficiency **(C)**, characteristic path length **(D)**, betweenness centrality of ROI #6 **(F)**, eccentricity of ROI #1 **(G)**, eccentricity of ROI #2 **(H)**, and eccentricity of ROI #16 **(I)**.

The results of statistical tests conducted on the temporal mean of MST parameters are shown in Table 2 and Figure 2. And the results of statistical tests conducted on the temporal variance of MST parameters are shown in Table 3 and Figure 3.

**Table 2:** The mean±SD of the temporal mean of MST parameters with significant group differences and associated *t* values.

	**mean (SD) of two groups**		***t*** **-value**
	**ASD group**	**TD group**	
Betweenness centrality of MST	0.6371 ± 0.0173	0.6401 ± 0.0159	−7.13^**^
Tree hierarchy	0.3379 ± 0.0232	0.3337 ± 0.0202	6.54^*^
Global efficiency	0.3405 ± 0.0054	0.3412 ± 0.0056	−3.98^*^
Betweenness centrality of ROI #1	0.2418 ± 0.1003	0.2670 ± 0.1026	−9.72^**^
Eccentricity of ROI #1	7.8138 ± 0.5652	7.6500 ± 0.6198	12.67^**^

* : *p* < 0.05;

** : *p* < 0.01.

**Table 3:** The mean±SD of temporal variance of MST parameters with significant group differences and associated *t* values.

	**mean (SD) of two groups**		***t*** **-value**
	**ASD group**	**TD group**	
Betweenness centrality of MST	0.0059 ± 0.0026	0.0054 ± 0.0023	7.94^**^
Tree hierarchy	0.0123 ± 0.0123	0.0099 ± 0.0096	8.60^**^
Global efficiency	4.0240 × 10^−4±^ 1.6335 × 10^−4^	3.7092 × 10^−4±^ 1.4217 × 10^−4^	7.83^**^
Characteristic path length	0.2492 ± 0.0672	0.2362 ± 0.0611	8.66^**^
Betweenness centrality of ROI #1	0.0444 ± 0.0116	0.0472 ± 0.0099	−10.46^**^
Betweenness centrality of ROI #6	0.0265 ± 0.0130	0.0245 ± 0.0134	7.96^**^
Eccentricity of ROI #1	3.7715 ± 0.8264	3.5710 ± 0.6860	13.10^**^
Eccentricity of ROI #2	3.6906 ± 0.7456	3.5430 ± 0.7025	7.78^**^
Eccentricity of ROI #16	3.6258 ± 0.7304	3.5008 ± 0.7187	7.44^**^

*: *p* < 0.05;

** : *p* < 0.01.

Compared with those in the TD group, the temporal mean of betweenness centrality of MST, global efficiency, and betweenness centrality of ROI #1 (i.e., aMPFC) was significantly lower in the ASD group, whereas the temporal mean of tree hierarchy and eccentricity of ROI #1 (i.e., aMPFC) were significantly higher in ASD group.

Compared with those in the TD group, the temporal variance of betweenness centrality of ROI #1 (i.e., aMPFC) was significantly lower in the ASD group, whereas the temporal variance of the following measures was significantly higher in the ASD group: betweenness centrality of MST, tree hierarchy, global efficiency, characteristic path length, betweenness centrality of ROI #6 (i.e., lTempP), eccentricity of ROI #1 (i.e., aMPFC), eccentricity of ROI #2 (i.e., PCC), and eccentricity of ROI #16 (i.e., rRsp).

### Associations between the temporal mean and variance of MST parameters and autistic symptom severity

By computing the correlation between the temporal mean of MST parameters and autistic symptom severity assessed by ADOS, we found the following (Table 4). First, the correlation between the temporal mean of the following two MST parameters and the ADOS_TOTAL score and ADOS_COMM score were significantly negative: betweenness centrality of ROI #4 (i.e., lTPJ) and degree of ROI #4 (i.e., ITPJ). Second, the correlation between the temporal mean of degree of ROI #9 (i.e., rTempP) and the ADOS_COMM score was significantly negative. Third, the correlation between the temporal mean of the eccentricity of ROI #4 (i.e., lTPJ) and both the ADOS_TOTAL score and ADOS_COMM score were significantly positive.

**Table 4:** The Pearson correlation coefficients between the temporal mean of MST parameters and autistic symptom severity.

	**ADOS scores**			
	**ADOS_COMM**	**ADOS_STEREO_BEHAV**	**ADOS_SOCIAL**	**ADOS_TOTAL**
Betweenness centrality of ROI #4	−0.3079^**^			−0.2287^*^
Degree of ROI #4	−0.3160^**^			−0.2344^*^
Degree of ROI #9	−0.1746^*^			
Eccentricity of ROI #4	0.1917^*^			0.1789^*^

Only significant correlation coefficients are presented.

* : *p* < 0.05;

** : *p* < 0.01.

By computing the correlation between the temporal variance of MST parameters and autistic symptom severity assessed by ADOS, we found the following (Table 5). First, the correlation between the temporal variance of the following two MST parameters and the ADOS_COMM score was significantly positive: global efficiency and eccentricity of ROI #4 (i.e., lTPJ). Second, the correlation between the temporal variance of degree of ROI #4 (i.e., lTPJ) and the ADOS_COMM score was significantly negative. Third, the correlation between the temporal variance of the following four MST parameters and the ADOS_SOCIAL score was significantly positive: betweenness centrality of ROI #7 (i.e., rTPJ), degree of ROI #15 (i.e., rpIPL), eccentricity of ROI #12 (i.e., lRsp), and eccentricity of ROI #14 (i.e., lHF^+^). Fourth, the correlation between the temporal variance of degree of ROI #4 (i.e., lTPJ) and the ADOS_Total score was significantly negative, whereas the correlation between the temporal variance of eccentricity of ROI #4 (i.e., lTPJ) and the ADOS_Total score was significantly positive.

**Table 5:** The Pearson correlation coefficients between the temporal variance of MST parameters and autistic symptom severity.

	**ADOS scores**			
	**ADOS_COMM**	**ADOS_STEREO_BEHAV**	**ADOS_SOCIAL**	**ADOS_TOTAL**
global efficiency	0.1739^*^			
betweenness centrality of ROI #7			0.2478^*^	
degree of ROI #4	−0.2674^*^			−0.2086^*^
degree of ROI #15			0.2383^*^	
eccentricity of ROI #4	0.2278^*^			0.1748^*^
eccentricity of ROI #12			0.1761^*^	
eccentricity of ROI #14			0.1927^*^	

Only significant correlation coefficients are presented.

* : *p* < 0.05.

## Discussion

In the current study, using the DCC technique and MST approach, the complex brain network within DMN was constructed for each sampling point (i.e., each TR). Then, network-level and node-level graph theory-based MST parameters were derived from MST of each sampling point. The temporal mean and variance of these MST parameters were further computed. We found that the two summary statistics of certain MST parameters were significantly altered in the ASD group, which suggests that the topographical configuration of the cortical network within DMN, along with its temporal dynamics, were significantly altered in the autistic brain. Moreover, using correlation analysis, we found that these alterations were significantly associated with the autistic symptom severity of persons with ASD.

### Altered MST parameters within DMN

Statistical tests conducted on the two summary statistics (i.e., temporal mean and variance) of MST parameters revealed some interesting results.

First, compared with those of the TD group, the temporal mean and variance of betweenness centrality of ROI #1 (i.e., aMPFC) were significantly smaller in ASD group, whereas the temporal mean and variance of eccentricity of ROI #1 (i.e., aMPFC) were significantly larger in ASD group. The relatively smaller temporal mean and variance of betweenness centrality of aMPFC suggest that in the autistic brain, the aMPFC is in a relatively inferior position since the less short paths are passing aMPFC. Moreover, compared with the autistic brain, the position of aMPFC varied drastically in a typically developing brain. The statistical results of eccentricity suggest that in the autistic brain, aMPFC locates away from the center of DMN and the distance from aMPFC to the other regions within DMN varies drastically. The aMPFC, especially the dorsal part, is supposed to be involved in social cognition processes, such as self-referential processes (33). The altered MST parameters related to aMPFC (i.e., reduced temporal mean and variance of betweenness centrality, enhanced temporal mean, and variance of eccentricity) may reduce the efficiency of information communication between aMPFC and other DMN regions. Considering the functions of aMPFC in the human cognition system, we supposed that the altered MST parameters related to aMPFC should result in the reduced efficiency of social information processing, such as the self-reference process in the autistic brain.

Second, as for ROI #2 (i.e., PCC), although none of the temporal means of MST parameters related to this brain region was found to be significantly altered, the temporal variance of eccentricity of PCC was significantly larger in persons with ASD. The PCC plays a crucial role in various cognitive functions, such as autobiographical memory (especially those involving friends and family members), and evaluation of valence of emotional stimuli (34, 35). Certain cognitive processes involving PCC were closely associated with the successful completion of social communication and social information processing. Jia et al. (15) have proved that significantly much lower and more volatile connections between PCC and other DMN regions were an important pathological feature of the ASD. Taken together with Jia et al. (15), the PCC of the autistic brain manifests the following characteristics: drastic variations of its distance to other DMN regions, and much lower and more volatile information exchange efficiency between PCC and other DMN regions. This will certainly reduce the efficiency and stability of information communication between aMPFC and other DMN regions. Considering the functions of PCC in social cognition, we supposed that the altered MST parameters related to PCC revealed in this study could impair the social information processing in the autistic brain and may contribute to the emergence of ASD-related symptoms.

Note that the reduced stability of information communication in social cognition related regions was supported by group comparison results of temporal variances of “position-related” parameters for ROI #6 (i.e., lTempP) and ROI #16 (i.e., rRsp). Table 3 shows the temporal variance of betweenness centrality of ROI #6 (i.e., lTempP) and the eccentricity of ROI #16 (i.e., rRsp) was significantly enlarged in autistic brains. These two cortical regions are crucial regions of the so-called “*social brain*” (36).

Third, statistical tests conducted on the temporal mean and variance of network-level MST parameters revealed that compared with those of the TD group, the temporal mean of betweenness centrality of MST was significantly smaller in the ASD group, whereas the temporal variance of betweenness centrality of MST was significantly larger in ASD group. Since the betweenness centrality of certain MST is defined as the maximum value of nodal betweenness centrality and could be used to describe the global network organization of MST, the above results suggest that the “load” of central nodes is much lower (i.e., the topographical configuration tends to be “link-like” topology) and varies drastically in the brain network of autistic brain (18). Considering the formula of the tree hierarchy of MST (i.e., the tree hierarchy of certain MST is the reciprocal of the betweenness centrality of this MST), the results of statistical tests conducted on the summary statistics of the tree hierarchy of certain MST could be easily verified. Moreover, these results were consistent with the results of global efficiency and characteristic path length: compared with those of the TD group, the temporal mean of global efficiency was significantly smaller, and the temporal variances of global efficiency and characteristic path length were significantly larger in the ASD group. Given the functional significance of these MST parameters, all these facts (i.e., altered topographical configuration, global efficiency, and characteristic path length) should lead to significantly lower efficiency and less robustness of information transfer within the DMN of the autistic brain, which should further affect the cognitive processes related with DMN (e.g., social information processing).

### Correlations between MST parameters and symptom severity

The correlation analysis between summary statistics of MST parameters and ADOS scores provide further evidence about the aberrant global network organization of DMN in the autistic brain.

First, as for ROI #4 (i.e., lTPJ), we found that the temporal mean of betweenness centrality of lTPJ and the temporal mean and variance of degree of lTPJ were significantly negatively correlated with the ADOS_COMM score and the ADOS_TOTAL score, whereas the temporal mean and variance of eccentricity of lTPJ were significantly positively correlated with the ADOS_COMM score and the ADOS_TOTAL score. These results indicated that “*the position of lTPJ within DMN*” could predict the autistic symptom severity of persons with ASD, and are highly consistent with previously published studies (15). For example, Jia et al. (15) found that the abnormal connections of lTPJ and the other regions within DMN were closely associated with the autistic symptom severity, especially the ADOS_COMM score. These results support the presumed functions of lTPJ (e.g., social communication development, joint attention, and theory of mind) and its role in the neuropathological mechanism of autism (37).

Second, the temporal mean of degree of ROI #9 (i.e., rTempP) was significantly negatively correlated with the ADOS_COMM score. The right temporal pole (rTemP) is crucial to understanding the mental state of others and the social communication (38). The relatively smaller degree of rTemP leads to the result that the information exchange efficiency related to rTemP is much lower and more paths are needed to communicate with this region, which may damage the social communication efficiency of persons with ASD.

Third, as for the temporal variances of MST parameters, the temporal variances of betweenness centrality of ROI #7 (rTPJ), eccentricity of ROI #12 (lRsp), eccentricity of ROI #14 (lHF^+^), and degree of ROI #15 (rpIPL), global efficiency was significantly positively correlated with the ADOS scores. These results indicate that the volatility of certain node-level/global level MST parameters could predict the pathological behavior and the defect in the social communication ability of these patients.

### Limitations and future directions

In the current study, the preprocessing pipeline has several separately conducted steps; each designed to remove a specific type or class of artifacts. This approach is named as “*the modular preprocessing approach*”, and has been commonly used in RS-fMRI studies (39). However, Lindquist et al. (39) showed that preprocessing steps performed at a later stage of the pipeline may potentially re-introduce artifacts that had previously been removed from the data in an earlier step, which may induce adverse effects on dFC estimations (39). They suggested that one way to avoid this problem was to simultaneously perform different preprocessing steps within an omnibus framework. In future studies, we need to perform the RS-fMRI preprocessing using the framework provided by Lindquist et al. (39), and test whether the preprocessing framework affects the main results revealed here.

Secondly, in the RS-fMRI datasets used here, the autistic symptom severity of persons with ASD was assessed *via* ADOS-module 3 or ADOS-module 4, and we did not consider the differences in the module of the ADOS. For the sake of completeness, we provided the Pearson correlation coefficients between the temporal mean/variance of MST parameters and autistic symptom severity assessed by ADOS-module 3/ ADOS-module 4 in Supplementary Tables S2–S5. The results presented in these four tables were not completely consistent with those displayed in Tables 4, 5 shown above. Although inconsistency could be clearly observed between these tables, the main conclusions of correlation analysis are still unchanged, i.e., the significantly lower and more volatile information communication within DMN is closely associated with the autistic symptom severity.

Thirdly, many graph theory-based complex network parameters which could be classified into the network-level parameters and node-level parameters were derived in our study. For these parameters, independent *t-*tests were conducted with groups (ASD group vs. TD group) as an independent variable, after controlling the effects of certain confounding variables. Then FDR procedure was applied for each node-level parameter, which may not fully control the multiple comparison problems. In the following statistical test, all the network-level and node-level parameters were included in the FDR procedure. We found that the *p*-values of the temporal means of tree hierarchy and global efficiency only reached a marginally significant level, and the statistical significance of other parameters did not change. This indicated that the FDR procedure could not significantly influence the conclusions revealed here.

Fourthly, we performed correlational analyses on the temporal mean and variance of all the parameters and did not merely focus on those showing significant between-group differences. This was because we believed that significant between-group differences revealed on certain parameters did not signify these parameters could predict the autistic symptom severity, and vice versa. Moreover, since the ADOS scores of some patients were invalid, the number of patients involved in group difference tests was larger than that involved in correlational analyses. All these arguments were supported by the fact that the parameters with significant correlation coefficients were not limited to the parameters with significant group differences.

Lastly, it is well known that ASD could affect the normal functioning of several brain networks, such as the DMN, salience network (SN), language network, and attention network (5, 7, 40–42). It is difficult to investigate the spatial organizations of all the networks within a single study, thus we focused on the DMN, which has been consistently observed in previous studies. In future studies, the dFCs and the MST characters of other cortical networks should be studied.

## Conclusion

In the current study, using the DCC technique and MST approach, the temporal dynamic features (i.e., temporal mean and variance) of topographical configuration of the cortical network within DMN of persons with ASD were investigated. The following conclusions could be derived from the statistical tests and correlation analyses. Firstly, in the autistic brain, aMPFC and PCC which are core regions of DMN locate away from the center of DMN. The information exchange efficiency between cortical regions within DMN is significantly lower and more volatile for patients with ASD. Secondly, the significantly lower and more volatile information communication within DMN is closely associated with autistic symptom severity. These results provide a novel insight into the possible neuropathological mechanism of ASD.

## Data availability statement

The original contributions presented in the study are included in the article/Supplementary material, further inquiries can be directed to the corresponding author.

## Ethics statement

The studies involving human participants were reviewed and approved by the Research Ethics Committee of 17 Institutions (i.e., California Institute of Technology/Carnegie Mellon University/Kennedy Krieger Institute/Ludwig Maximilians University Munich/NYU Langone Medical Center/Olin Center, Institute of Living at Hartford Hospital/Oregon Health and Science University/San Diego State University/BCN NeuroImaging Center, University Medical Center Groningen/Stanford University/Trinity Centre for Health Sciences/University of California, Los Angeles/University of Leuven/University of Michigan/University of Pittsburgh/University of Utah/Yale Child Study Center). Written informed consent to participate in this study was provided by the participants' legal guardian/next of kin.

## Author contributions

HJ and XW initiated and analyzed the data and wrote the manuscript. ZW and EW wrote and revised the manuscript. All authors have read and approved the final published manuscript.

## Funding

This work was supported by Post-funded Project of the National Social Science Fund under Grant 20FJKB005, Henan Province Philosophy and Social Sciences Outstanding Scholars Project under Grant 2018-YXXZ-03, the Philosophy and Social Sciences Planning Project of Henan Province under Grant 2020BJY010, Postgraduate Cultivating Innovation and Quality Improvement Action Plan of Henan University under Grant SYLYC2022039, and Henan University Philosophy and Social Science Innovation Team under Grant 2019CXTD009.

## Conflict of interest

The authors declare that the research was conducted in the absence of any commercial or financial relationships that could be construed as a potential conflict of interest.

## Publisher's note

All claims expressed in this article are solely those of the authors and do not necessarily represent those of their affiliated organizations, or those of the publisher, the editors and the reviewers. Any product that may be evaluated in this article, or claim that may be made by its manufacturer, is not guaranteed or endorsed by the publisher.
