# Supplementary material for: Aberrant dynamic minimal spanning tree parameters within default mode network in patients with autism spectrum disorder
Source: Front Psychiatry. 2022 Sep 15;13:860348. doi: 10.3389/fpsyt.2022.860348 (PMC9524021; doi:10.3389/fpsyt.2022.860348)
Supplement: Supplementary file 1 [file Table_1.DOCX]

Supplement to: “Aberrant dynamic minimal spanning tree parameters within default mode network in patients with autism spectrum disorder”

**Huibin Jia^1,2^** **^†^, Xiangci Wu^1,2 †^, Zhiyu Wu^3 †^ and Enguo Wang^1,2*^**

^1^Institute of Psychology and Behavior, Henan University, Kaifeng, 475004, China

^2^School of Psychology, Henan University, Kaifeng, 475004, China

^3^Huaxian People’s Hospital of Henan Province, Anyang, 456400, China

***Correspondence:**

Enguo Wang

enguowang@126.com

^†^These authors have contributed equally to this work and share first authorship

**Table S1** The demographic and basic clinical characteristics of ASD group and TD group of 17 institutions (i.e., California Institute of Technology/Carnegie Mellon University/Kennedy Krieger Institute/Ludwig Maximilians University Munich/NYU Langone Medical Center/Olin Center, Institute of Living at Hartford Hospital/Oregon Health and Science University/San Diego State University/BCN NeuroImaging Center, University Medical Center Groningen/Stanford University/Trinity Centre for Health Sciences/University of California, Los Angeles/University of Leuven /University of Michigan/University of Pittsburgh/University of Utah/Yale Child Study Center) used in current study. Note: IQ = intelligence quotient; Mean_fd = mean framewise displacement; Perc_fd = percent framewise displacement greater than 0.2 mm.

|  | ASD Group | TD Group |
| --- | --- | --- |
| IQ (Mean±SD) | 106.47±16.08 | 111.28±12.36 |
| Age (Mean±SD) | 16.31±7.03 years | 16.43±6.93 years |
| Mean_fd (Mean±SD) | 0.08±0.04 mm | 0.07±0.04 mm |
| Perc_fd (Mean±SD) | 6.46%±6.58% | 4.60%±5.76% |
| Sex (number of participants) | 299 (male), 44 (female) | 347 (male), 81 (female) |
| ADOS_TOTAL (Mean±SD) | 11.66±3.79 | N/A |
| ADOS_COMM (Mean±SD) | 3.82±1.49 | N/A |
| ADOS_SOCIAL (Mean±SD) | 7.95±2.76 | N/A |
| ADOS_STEREO_BEHAV (Mean±SD) | 2.45±1.36 | N/A |

**Table S2** The Pearson correlation coefficients between the temporal mean of MST parameters and autistic symptom severity assessed by ADOS (module 3). Only significant correlation coefficients were presented.

|  | ADOS scores | | | |
| --- | --- | --- | --- | --- |
|  | COMM | STEREO_BEHAV | SOCIAL | TOTAL |
| betweenness centrality of ROI #4 | -0.3388^**^ |  |  | -0.2459^*^ |
| betweenness centrality of ROI #13 |  |  |  | -0.2214^*^ |
| degree of ROI #4 | -0.3331^*^ |  |  | -0.2485^*^ |
| degree of ROI #9 | -0.2557^*^ |  |  |  |
| degree of ROI #13 |  | -0.2412^*^ |  | -0.2432^*^ |

**Table S3** The Pearson correlation coefficients between the temporal mean of MST parameters and autistic symptom severity assessed by ADOS (module 4). Only significant correlation coefficients were presented.

|  | ADOS scores | | | |
| --- | --- | --- | --- | --- |
|  | COMM | STEREO_BEHAV | SOCIAL | TOTAL |
| betweenness centrality of ROI #12 | -0.3920^**^ |  |  | -0.3013^*^ |
| degree of ROI #12 | -0.3934^*^ |  |  | -0.3061^*^ |

**Table S4** The Pearson correlation coefficients between the temporal variance of MST parameters and autistic symptom severity assessed by ADOS (module 3). Only significant correlation coefficients were presented.

|  | ADOS scores | | | |
| --- | --- | --- | --- | --- |
|  | COMM | STEREO_BEHAV | SOCIAL | TOTAL |
| betweenness centrality of ROI #7 |  |  | 0.2306^*^ |  |
| degree of ROI #4 | -0.2632^*^ |  |  |  |
| degree of ROI #6 | 0.2297^*^ |  |  |  |
| degree of ROI #7 | 0.2152^*^ |  | 0.2149^*^ |  |
| degree of ROI #13 | -0.2164^*^ |  |  |  |
| degree of ROI #15 |  |  | 0.2859^**^ |  |

**Table S5** The Pearson correlation coefficients between the temporal variance of MST parameters and autistic symptom severity assessed by ADOS (module 4). Only significant correlation coefficients were presented.

|  | ADOS scores | | | |
| --- | --- | --- | --- | --- |
|  | COMM | STEREO_BEHAV | SOCIAL | TOTAL |
| global efficiency |  |  | 0.3473^*^ |  |
| betweenness centrality of ROI #3 | -0.3173^*^ |  |  |  |
| degree of ROI #12 | -0.3525^*^ | -0.3244^*^ |  | -0.3591^*^ |
| degree of ROI #13 |  |  | 0.3017^*^ |  |
| degree of ROI #15 |  |  | 0.2859^**^ |  |
| eccentricity of ROI #10 |  |  | 0.3871^*^ |  |
| eccentricity of ROI #14 |  |  | 0.5112^**^ |  |
| eccentricity of ROI #15 |  |  | 0.4034^**^ |  |
